# Supplementary material for: Selection Response Due to Different Combination of Antagonistic Milk, Beef, and Morphological Traits in the Alpine Grey Cattle Breed
Source: Animals (Basel). 2021 May 8;11(5):1340. doi: 10.3390/ani11051340 (PMC8151928; doi:10.3390/ani11051340)
Supplement: Supplementary file 1 [file animals-11-01340-s001.zip › animals-1189961-supplementary.pdf]

Supplementary Material 1 Genetic (above the diagonal), and phenotypic (below the diagonal) correlations among milk traits, SCS, morphological and beef traits analyzed. Values reported are means and HPD of the marginal posterior density's region of estimates at 5 and 95% (within brackets).

TRAIT[1]	MY	FY	PY	SCS	F2<br>UV	F3<br>UC	F7<br>RL	HT	RM	ADG	SEUROP	CY
**MY**		0.758<br>(0.732<br>0.781)	0.845<br>(0.829<br>0.860)	0.069<br>(-0.001<br>0.141)	0.330<br>(0.235<br>0.421)	-0.448<br>(-0.546 -<br>0.354)	0.060<br>(-0.066<br>0.182)	-0.091<br>(-0.191<br>0.011)	-0.458<br>(-0.547 -<br>0.365)	-0.071<br>(-0.362<br>0.187)	-0.240<br>(-0.491<br>0.001)	-0.156<br>(-0.363<br>0.051)
**FY**	0.768<br>(0.752<br>0.802)		0.824<br>(0.804<br>0.844)	0.067<br>(-0.008<br>0.144)	0.286<br>(0.185<br>0.383)	-0.326<br>(-0.429 -<br>0.224)	0.045<br>(-0.086<br>0.175)	-0.136<br>(-0.241 -<br>0.033)	-0.413<br>(-0.514 -<br>0.316)	-0.092<br>(-0.339<br>0.145)	0.029<br>(-0.196<br>0.233)	-0.103<br>(-0.390<br>0.140)
**PY**	0.905<br>(0.850<br>0.960)	0.766<br>(0.244<br>0.247)		0.088<br>(0.014<br>0.164)	0.289<br>(0.188<br>0.388)	-0.423<br>(-0.521 -<br>0.322)	0.099<br>(-0.031<br>0.228)	-0.169<br>(-0.274 -<br>0.062)	-0.397<br>(-0.493<br>0.299)	-0.066<br>(-0.280<br>0.139)	0.175<br>(-0.102<br>0.433)	-0.156<br>(-0.385<br>0.073)
**SCS**	-0.149<br>(-0.250 -<br>0.050)	-0.08<br>(-0.120 -<br>0.020)	-0.111<br>(-0.195 -<br>0.060)		0.246<br>(0.120<br>0.369)	0.149<br>(0.009<br>0.284)	0.190<br>(0.005<br>0.240)	-0.109<br>(-0.223<br>0.004)	-0.158<br>(-0.274 -<br>0.039)	-0.184<br>(-0.452<br>0.043)	-0.008<br>(-0.219<br>0.182)	-0.259<br>(-0.475 -<br>0.054)
**F2-UV**	0.240<br>(0.211<br>0.244)	0.172<br>(-0.080 -<br>0.051)	0.211<br>(-0.119 -<br>0.085)	-0.001<br>(-0.006<br>0.028)		-0.208<br>(-0.340 -<br>0.073)	0.097<br>(-0.062<br>0.260)	0.129<br>(-0.005<br>0.259)	-0.319<br>(-0.441 -<br>0.190)	-0.121<br>(-0.395<br>0.144)	-0.351<br>(-0.536 -<br>0.129)	-0.359<br>(-0.736 -<br>0.040)
**F3-UC**	-0.122<br>(-0.137 -<br>0.101)	-0.067<br>(-0.010<br>0.028)	-0.104<br>(-0.300<br>0.037)	0.012<br>(-0.006<br>0.028)	0.030<br>(-0.120<br>0.054)		0.098<br>(-0.067<br>0.255)	0.079<br>(-0.057<br>0.209)	0.346<br>(0.224<br>0.463)	-0.128<br>(-0.444<br>0.157)	0.067<br>(-0.210<br>0.334)	0.061<br>(-0.258<br>0.381)
**F7-RL**	0.02<br>(0.001<br>0.038)	0.014<br>(-0.213<br>0.147)	0.021<br>(-0.238<br>0.157)	0.012<br>(-0.023<br>0.056)	0.033<br>(-0.326<br>0.224)	0.01<br>(-0.243<br>0.344)		0.075<br>(-0.053<br>0.197)	-0.324<br>(-0.460 -<br>0.183)	0.148<br>(-0.176<br>0.426)	-0.156<br>(-0.457<br>0.157)	-0.159<br>(-0.573<br>0.213)
**HT**	-0.02	-0.012	-0.023	-0.022	0.007	0.021	0.009		0.075	-0.189	0.208	0.171

	(-0.093 - 0.002)	(-0.072 0.035)	(-0.135 0.070)	(-0.113 0.011)	(-0.036 0.014)	(-0.040 0.106)	(-0.014 0.040)		(-0.053 0.197)	(-0.477 0.081)	(-0.051 0.463)	(-0.129 0.481)
**RM**	-0.134 (-0.349 - 0.265)	-0.079 (-0.066 0.006)	-0.086 (-0.100 - 0.013)	0.001 (-0.095 - 0.012)	-0.120 (-0.114 - 0.227)	0.128 (-0.006 0.159)	-0.177 (-0.262 - 0.151)	0.085 (0.031 0.180)		0.656 (0.343 0.909)	0.798 (0.556 0.981)	0.849 (0.540 0.991)
**ADG**	-0.014 (-0.069 0.036)	-0.018 (-0.045 0.053)	-0.015 (-0.021 0.085)	-0.046 (-0.110 0.093)	-0.034 (-0.437 0.097)	-0.037 (-0.169 0.264)	0.043 (-0.313 0.111)	-0.064 (-0.105 0.405)	0.582 (0.408 0.719)		0.839 (0.594 0.996)	0.545 (0.156 0.977)
**SEUROP**	-0.057 (-0.122 0.000)	0.006 (-0.088 0.031)	0.034 (-0.106 0.022)	-0.002 (-0.257 0.031)	-0.133 (-0.211 - 0.014)	0.025 (-0.094 0.129)	-0.052 (-0.151 0.068)	0.087 (-0.049 0.183)	0.276 (0.184 0.338)	0.621 (0.614 0.741)		0.928 (0.831 0.990)
**CY**	-0.047 (-0.219 0.034)	-0.024 (-0.088 0.031)	-0.041 (-0.106 0.022)	-0.07 (-0.257 - 0.031)	-0.109 (-0.211 - 0.014)	0.019 (-0.094 0.129)	-0.041 (-0.151 0.068)	0.06 (-0.049 0.183)	0.241 (0.184 0.338)	0.545 (0.380 0.994)	0.825 (0.780. 1.00)	
